# Medical Quacks, Their Methods and Dangers

**Published:** 1905-10

**Authors:** Champe S. Andrews

**Affiliations:** New York


### Medical Quacks, Their Methods and Dangers.

BY CHAMPE S. ANDREWS, ESQ., New York.

[From *Critic and Guide.*]

IN November, 1808, one Samuel Thompson came into Beverly, Mass., and, proclaiming his ability to heal the sick without fail, declared that the country was being ruined by the regular physician. As his experience with the credulous warranted him in believing, there were those, even in enlightened Massachusetts, who took him at his word. Patients flocked to him by the score. Ezra Lovett, Jr., finding himself confined to bed by a cold, sent for the now celebrated Thompson. He came, and, I have no doubt, received his pay in advance. "Dr." Thompson had the patient's room heated as hot as the stove could make it. And the report of the case, found in 6 Mass., 134 *et seq.*, tells us in great detail of the healer's methods:

He then placed the feet of the deceased, with his shoes off, on a stove of hot coals, and wrapped him in a thick blanket covering his head. In this situation he gave him a powder in water, which immediately puked him. Three minutes after he repeated the dose, which in about two minutes operated violently. He again repeated the dose, which in a short time operated with more violence. These powders were all given within the space of half an hour, the patient in the meantime drinking copiously of a warm decoction which the "Dr." called "coffee."

The "coffee" administered was a decoction of marsh-rosemary, mixed with the bark of bayberry bush. The powders called by the "Dr." "well-my-gristle" and "ram-cats," which the prisoner said he chiefly relied upon in his practice, and which was the emetic so often administered by him to the patient was the pulverised plant trivially called "Indian tobacco," four grains of which was a powerful puke and cathartic.

The report goes on to relate that the learned physician ordered his patient to sleep in a warm bed, "where he lay in a profuse sweat all night." The next (Tuesday) afternoon more powders of "well-my-gristle" and "ram-cats" were administered in quick succession, washed down by "coffee." On Wednesday the patient's face and hands were rubbed with rum and he was given fifteen minutes of nature cure by exposure to the January winds in the open air. As a reward more ram-cats were washed down by more coffee. With variations this heroic treatment continued until the following Monday, when the "Dr." was specially summoned as the patient insisted he was dying.

Pearlash, "coffee" and "well-my-gristle" were administered in quick succession and the patient was asked how far the medicine had gotten down. The patient, laying his hand on his heart, answered "*here*" when the "doctor" observed that the "medicine would soon get down and unscrew his vitals." It did. Between nine and ten o'clock convulsions set in, the patient lost his reason, and on the next day, after one week's treatment, Ezra Lovett, Jr., died.

The object of this article is not to point out the history and present status of the law governing illegal practice or the rules governing the trial of a quack charged with manslaughter or murder. Its object is to point out by concrete example the great dangers resulting from the practice of the quack, and the crying necessity for the enforcement of the laws designed to protect the public against these dangers.

### THE MODERN CHARLATAN.

As education has become more generally diffused and science more advanced, the medical charlatan has changed his methods to correspond with the new order of things. The modern endeavor is to imitate the true scientific methods, and, with "a little learning" to make louder claims to specialised knowledge and advanced methods than does the regular practitioner. Strange as it may seem to know that "Dr." Thompson could have deceived anybody even a hundred years ago, the helpless and afflicted in these opening years of the Twentieth Century are victimised more completely and more artistically than was Ezra Lovett, Jr. Indeed the modern methods of advertising, the rapidity with which the false claims of the charlatan can be disseminated, and the ease with which the post-office and newspaper penetrate every section of the country have given the fakir of to-day a field of operation that "Doctor Thompson" never dared to contemplate.

### THE HELPLESSNESS OF THE CHARLATANS' VICTIMS.

As one learns in detail of the methods and dangers of the modern quack, there is at first a tendency to believe that the credulity of mankind is growing alarmingly greater, but a deeper study of the subject shows that the credulity upon which the charlatan relies, is the credulity that arises from weakened powers of resistance, from disordered minds, and from the mirage that such minds see mirrored in clear sky of hope. The victim of the medical mountebank, by reason of his susceptibilities and infirmities, is in a class to himself and should have the especial care and protection of the state.

The gambler for the most part takes away from his victims only that which the victim can again recover by prudence and industry. The political mountebank may lead the people away from the honest path for a time, but the wandering in time generally proves self-corrective. The religious fraud exists for a season with fashionable following, but in the end he talks to empty benches. The victims of the medical charlatan, however, have nothing like the same chance to retrieve their false steps as the victims of the other fakirs mentioned. The patients of the quack are either hypochondriacs, who remain eternally ill in their own diseased imaginations and furnish a never-failing harvest for the scythe of the charlatan, or else by reason of genuine maladies their power of resistance has gone and the illusions of hope created by marvelous claims and extravagant promises cause them to grasp at every cure-all just as the drowning man grasps at a straw.

In the City of New York, the danger of the charlatan is seen at its worst. The thickly settled neighborhoods where the ignorant foreigner resides are hot-beds of unlicensed and illegal practitioners, while the advertisements unblushingly accepted by a majority of New York newspapers, with their enormous circulation both in and out of the city, furnish the widest possible field for the modern quack, whose very life lies in newspaper publicity.

### MEDICAL SOCIETY OF THE COUNTY OF NEW YORK.

The State of New York, in 1806, perceived the necessity of protecting the public against empirical methods in medicine, and organised in that year, by a special charter which continues to this day, the Medical Society of the State of New York with its branch society in each county of the state. The act was entitled "An Act to Regulate the Practice of Physic and Surgery."

In later years these various county societies have, to a greater or less extent, been the only organised bodies standing between the charlatan and his victim. Theoretically, the Police Department and the District Attorney's Office are charged with the prosecution of all offenses, but where life is as complex as in the City of New York, and where the regular departments of the government charged with the suppression of crime are busy with crimes of murder, arson, larceny and the more heinous crimes generally, it is found impossible in practice for these departments to suppress the unlicensed practitioners of medicine. The presence of a body composed of the organized medical profession engaged, with the sanction of the state, in suppressing not only the criminal practitioner who is licensed, but also the practitioner without a license, is a salutary force in the community and commands, I believe, the respect and confidence of the public and acts as a constant deterrent to the wrong-doer.

The Medical Society of the County of New York for a great many years has maintained a separate legal bureau for the suppression of the unlicensed and criminal practitioner. A staff of well-trained detectives and agents are constantly investigating complaints and procuring evidence where the law is being violated. The method of procuring evidence by detectives is unavoidable because the victims of the charlatan are most reluctant to appear in court as complaining witnesses. It generally brings humiliation and disgrace upon them, exposes their maladies, real and imaginary, to the public, and is in itself a confession that they have been the willing dupes of preposterous frauds.

By means of the efforts of the Medical Society of the County of New York hundreds of cases involving a violation of the Public Health Law have been presented to the criminal courts in New York City. Since 1900, the legal department of that society has been in charge of the writer, and it has been his duty to institute these prosecutions, and by the courtesy of the District Attorney of New York County, to present the evidence to the various County Magistrates and to the Court of Special Sessions.

*The charlatans operating in New York City* may be divided into the following classes: (1) Those who prey upon the consumptive poor; (2) Advertising "Specialists for men only;" (3) Occultists; (4) Nature Cure and Water Cure doctors; (5) Midwives; (6) "Osteopaths," "Osteotherapists," "Somatopathists," "Mechano-Neural-Therapists," "Vitopaths," and scores of others of similar ilk, whose chief object seems to be to seek refuge in an imposing title. To these classes must be added the men and women who assume the names of deceased physicians and who practice under their diplomas. Others still boldly use their own names and falsely claim to be members of the medical profession, in the hope that in a city of four millions of people and many thousands of doctors, they may be overlooked and left unmolested.

The cases now to be mentioned, as illustrating the actual methods of each of the classes mentioned, were all presented to the courts in New York City by the Medical Society of the County of New York.

"Occultists."—The fakir who poses as a master of occult mysticism and esoteric philosophy finds a great many lovers of the mysterious willing to listen to his blandishments. In May, 1901, a complaint was received against "Doctor A. de Sarak," of 413 West Fifty-Seventh Street. An agent of the society called upon the "doctor" and was received with oriental salaams and greetings. The agent and the learned occultist soon got down to business and the doctor's inevitable card was presented to his supposed client. It read as follows:

"Doctor A. de Sarak, Oriental Scientist, General Delegate of the Scientific Academy of 'Sauveteur' of Paris, Member of the Oriental Society of Thibet and Calcutta, etc. Consultations of Oriental Sciences, on Magnetism, Double Vision, Concentration, Telepathy and Palmistry. Every day, except Sundays, bet. 3 and 5 P. M. 413 West Fifty-seventh street, New York. Consultations by correspondence. English, French, Italian, Spanish, Portuguese and Russian Spoken."

This wonderful assortment of methods and languages, together with the impressive display of decorations, medals and ribbons, soon led to a contract. The patient complained of headache, chills and sleeplessness. The doctor produced an ophthalmoscope and thermometer, and after making a few mysterious gestures and uttering weird incantations, pronounced the trouble as "nervous neurasthenia." He recommended a drug of unpronounceable name, and suffered the patient to depart after extracting $3 for the treatment. The "doctor" tried his case most vigorously —in the newspapers—but in the Court of Special Sessions pleaded guilty and without a murmur paid a fine of $50. A year or two afterward, clad in the same gorgeous attire, the "doctor" appeared for a brief season at one of the fashionable hotels in the city, where many respectable people accepted him at his own valuation. But his former experience served him in good stead, and he soon folded his tents and silently stole away.

The feminine variety of this interesting form of fakirs is illustrated in "Madame Marcella Bryan," convicted on January 16, 1903. I shall let her tell her own story. Scorning the services of a lawyer, she addressed the learned judge as follows:

I am a maker and seller of magic potions. My chief production of mysterious alchemy is 'Sympathy Water.' This one washes with and immediately does it take the devil out of one. That is why I have the great reputation and why Madame Brvan is known the world over. Shall I tell you more? Yes? I am also a clairvoyant, and I know magic and am of the mysterious spirit. I help people in business trouble. I help the maiden, the wife, the husband, the lover in the troubles of the heart. I can do anything; but practice medicine—never.

But alas for this wonderful healer, Mr. Justice Olmstead, speaking for the Court, pronounced her guilty, and forthwith she took her place along the great multitude of persecuted scientists.

MIDWIVES.—The midwife has no recognised status in the laws of the State of New York. As long as she confines her activities to the sphere that custom for generations has alloted to her, it has not been the policy of the medical profession to disturb her. But as a matter of fact, there are no bolder criminal practitioners in New York City than are to be found among the midwives. It is safe to say that a large majority of them will undertake, without hesitation, to perform criminal operations or prescribe drugs for a criminal purpose. Over fifty convictions for such offenses have been secured recently by the Medical Society. Many deaths occur every year as a result of the ignorance and criminal practice of the midwives of New York.

In the last ten days we brought to the attention of the Courts, Yetta Prince, an East Side midwife. She undertook to relieve a poor woman of sterility. Sixteen treatments resulted in a double pyosalpinx, followed by an operation and the removal of both ovaries and the tubes. She was sentenced to a term of imprisonment.

UNWARRANTED USE OF TITLE "DOCTOR."—"DIPLOMA MILL." A most interesting fraud is the man who boldly assumes the title of doctor without any claims whatever to a medical education. The most brazen and successful practitioner of this kind that the Society has ever dealt with is "Dr." Charles Conrad, convicted April 21, 1904.

On April 7 he wrote to Dr. J. McPherson Scott, Secretary of Board of Medical Examiners for the State of Maryland, asking permission to register as a physician in that state. In his letter he said: "I am not licensed in any state, but I hold a diploma from the 'Vetus Academia Physio Medica.'" He asked to be permitted to register in Maryland without going to the trouble of appearing there in person.

This letter was sent to the regents of the University of the State of New York, who in turn forwarded it to the Medical

Society of the County of New York. Upon investigation we ascertained that the defendant was formerly a Norwegian sailor with no pretense to medical knowledge. The "Vetus Academia Physio Medica" was a college of which he was the president and faculty. He printed elaborate diplomas on parchment, couched in wonderful Latin, but he was willing to sell one to every applicant for $500.

At 56 West Sixty-fifth Street, he was conducting the Platen Institute, with two branches in Harlem, each branch in charge of a "graduate" of his celebrated school, and supplied with a staff of nurses. Almost any form of treatment desired was administered. This man went so far as to have a bill introduced into the last Senate of New York State (Bill No. 983, March 21, 1904) entitled a bill "To define and regulate the practice of Osteotherapy." Of course, "Dr." Conrad was the founder of the great healing science, osteotherapy.

His card surpasses criticism and description.

Dr. C. Conrad, Founder, President and Medical Director of Vetus Academia Physio Medica (Inc.), Founder, President and Director in Chief of the Platen Institute (Inc.), Lecturer on Psychology and Physiology in the Old Physio Medical College; Founder of Osteotherapy, Demonstrator and Lecturer on Osteotherapy at Platen Institute; Founder and President of New York Society of Osteotherapeutic physicians; Founder and Editor-in-Chief of the Twentieth Century Journal in Osteotherapy. Vice-President of the American Association of Physicians and Surgeons. Telephone, 2204 Columbus. By Appointment Only. Office, 56 West Sixty-fifth Street, New York, N. Y.

None of these institutions existed save in his own vivid imagination. A similar case is that of Joseph Rohrer, convicted February 17, 1902, for practising without registration and on May 19, 1904, for using the title "M.D." as President of "International Massage and Movement Cure Institute," which he advertised in over fifty papers and described in an elaborate book of 61 pages, containing many reproductions of the learned doctor in various professional poses.

Closely akin to this form of fraud is the man who assumes the name of some licensed physician who has recently died or removed from the community. The most recent conviction of this sort was a Russian who was a former valet to a London physician, and who, at the latter's death, stole his diploma and attempted to practise in this country.

In the east side district of New York, there have been several instances of the diploma of a deceased physician bringing at auction more than all the other assets of the estate. An advertisement recently appeared in a New York newspaper offering $1,000 for the diploma of a registered physician in this state who had not been dead over five years.

PRACTITIONERS OF OSTEOPATHY AND THE ART OF MANIPULATION.—The "Osteopath," the "Mechano-Neural Therapist," the "Somatopath," and other practitioners who attempt to cure disease by manipulation stand in a class by themselves. The cult of osteopathy has made considerable progress in this country within the past ten years in States where the standard of medical education is low. Many of the states of the Union have legalised the practice, but in New York and New Jersey and the Eastern states generally, where a high standard of medical education is maintained, the legislatures have persistently refused to grant them any recognition.

The osteopath claims that disease results from a disarrangement of the anatomy, and he includes in this disarrangement of the organs and circulation of the body, particularly nervous circulation as a result of local disturbances and influences. He insists that the proper method for cure is to remove the inflammation or dislocation by manipulation, massage, and kindred methods without the use of drugs.

A small percentage of these practitioners are, no doubt, sincere in their claims but a great herd of them throughout the United States take up this system of healing because of the ease with which it may be mastered and the short course of study necessary to obtain a diploma. A four years' course in a medical college is now requisite in the State of New York, whereas an osteopathic degree in other states may be secured in from one to two years, or less, according to the character of the institution.

WHAT IS THE "PRACTICE OF MEDICINE?"—This cult is responsible for the prevalent belief that to practise medicine is to give drugs. The courts of original jurisdiction in New York State have uniformly held that the practice of medicine is the practice of the healing art generally, and anyone who undertakes to diagnose and cure disease is practising medicine within the meaning of the statute. None of the higher courts have yet passed upon this question, and until such a decision is obtained, the law on this point will be in confusion. One of the resulting harms from the presence of this system of healing in a community is the encouragement it gives to unlicensed practice of all sorts where drugs are not administered. The practitioner who administers only electrical treatment or baths or massage or magnetic healing is as much entitled as the osteopath to claim that he is not practising medicine because he gives no drugs. All of these practitioners unhesitatingly undertake to cure the most serious forms of diseases, and hope to escape the penalty of the law because they avoid the use of medicine. If such a decision ever comes, the most appropriate comment on it that could be made is the comment made in 1806 in the report of the Thompson case previously referred to and found in 6 Mass., 134.

It is to be exceedingly lamented that people are so easily persuaded to put confidence in these itinerant quacks, and to trust their lives to strangers without knowledge or experience. If this astonishing infatuation should continue, and men are found to yield to the imprudent pretensions of ignorant empiricism, there seems to be no adequate remedy by a criminal prosecution without the interference of the legislature.

The enlightened medical profession of today makes use of all rational methods of healing, including suggestion, manipulation, massage and electricity. It is the height of injustice in view of this fact to say to the regular physician that he must acquire a knowledge of the healing art by a four years' course of study and at the same time permit the osteopath and all of his ilk who follow a short cut to the healing art to practise their cults without molestation or conditions as long as they do not administer drugs. As was said in a recent editorial in the New York *Times* "A course in medicine and surgery is expensive, and it takes a lot of time, while a varied assortment of pseudo-religious and pseudo-philosophic phrases can be learned in a few days by any man or woman with a disinclination for honest work."

WATER CURE.—The "practitioner" who pins his faith in water, styles himself by various titles ranging from Nature Cure to Balneotechnic. The "latest specimen" of this variety is "Dr." Charles F. Starken, who carried on a thriving business at Fifty-second Street and Broadway before his career was cut short by the unsympathetic agents of the society. His card read as follows:

C. F. Starken, Physician of Natural Cure and Balneotechnic, cures all kinds of Diseases without Medicine or instrument. Gives all kinds of Massage and Heat Gymnastics, Magno-Electric and Hydropathic Treatments, Also all kinds of Cure-Baths, Herbs, Mineral, Sulphur, Iron, Lithion, Pine-needles, Aromatic and all Medicated baths. Specially for Blood purifying and good Complexion. Moussir, Steam, Hot Air, Vapor and Astringent Baths. For Males, Females, and Children. Prices Liberal. Dr. C. F. Starken, Consultation: 9-10 A.M.; 2-3 P. M., 6-7 P. M. Fifty-second Street and Broadway, New York.

After the "Doctor's" methods had been called to the attention of the Medical Society by the New York Society for the Prevention of Cruelty to Children, two agents of the society, both women, sought the professional services of the celebrated watercure specialist. They described a variety of symptoms and pains, but the doctor was equal to the emergency and his diagnosis showed several serious complications. He scorned to cure them with drugs, but proceeded to administer a variety of baths, followed by massage. One of the agents in Court described her experience as follows:

I told him I was troubled with fainting spells and pains in my arms and shoulders. He asked me to remove my clothes, which I refused to do. He examined my chest. He took hold of both of my arms. I do not know what he did, but I screamed, it hurt me so. I said, what do you think is the matter with me? He said I had stagnation of the lungs; perhaps he meant congestion of the lungs. He said I was troubled with neuritis.

In some unimportant particulars, the learned gentleman's testimony contradicts the testimony of the People, but I am sure his own story is more interesting than any that could be framed for him. This is his story as taken from the minutes of his trial, March 31, 1904:

I said, what can I do for you? She said she would like a treatment. I said what is the matter with you? I said I do not want to give any treatment until I know what is the matter with you. I said this is not a Turkish bath. I said you might drop down here dead. She said she had a pain here and a pain there, and much pain all over. I said, is there a name for that and a that for that; I said there is a name for every kind of disease; if an organ is affected we call it that name, and if this organ is affected we call it that name. She said she would like to take a treatment and asked how much it was. I said $3. She left. She said she would come again. My nurse let her in again. She said she wanted to take my treatment. I said, wait a moment, and a woman will come and help you along. My nurse came along and brought her into the steam bath. I said, are you hot here? She said, yes it is quite hot. I felt of her temples, how her temperature was. Then the nurse took her to a bath and there were pine needles in it to clean the skin. Then I gave her a douche. My nurse brought her back to the table and wiped her off. When I was through with the other people I went back to the room where she was. I started to massage her. I must test the muscles—.

The Court.—Don't give us a lecture on that.

The witness continued. I gave her a rub. She asked, What is the matter with me? I said there is a lot of uric acid in you. She said, Can you give me any medicine? I said no, I give no medicines at all. I said I did not think the treatment would do her much good, because she was too timid.

Q. Was anything said about the cirrhosis of the liver?

A. No, sir. She said she had pains. And I said if she had a pain there it could not be cirrhosis of the liver.

His discourse did not have the desired effect, for Mr. Justice Wyatt, speaking for the Court, said:

The sentence of the Court is that the defendant shall pay a fine of $100, and in default of payment that he shall stand committed to the City Prison for a term of thirty days. You will have to stop this business.

FRAUDS WHO PRACTICE ON THE CONSUMPTIVE POOR.—Of all invalids the consumptive seems to believe more firmly than all others in his ultimate success in combating disease. The unscrupulous quack takes pitiless advantage of this weakness of the consumptive, and in his advertisements makes greater claims and pretensions perhaps than any other medical fraud.

In support of this assertion, I quote from a statement from S. A. Knopf, M. D., of New York City, to whom the "International Congress to Combat Tuberculosis as a Disease of the Masses," at Berlin, awarded the international prize for the best essay, on July 31, 1900. In a statement to the Charity Organisation Society of New York, Dr. Knopf says:

How many poor consumptives have lost their last little reserve fund by giving everything they had for a dozen bottles of the "sure and quick cure" only those who come in contact with them know. How unscrupulous some of these charlatans are in their method of procuring certificates of cure, which they then publish as bait to the unfortunate help-seeking sufferer, is something which can hardly be believed. Let me tell you of one instance: A poor woman in the last stages of consumption came to me seeking advice. When asked for the name of her former medical attendant, she confessed that she had been treated for a number of weeks by a quack concern, and now, her means being exhausted she was made to understand that they would not continue to treat her unless she would give them a certified testimonial that she had been thoroughly cured of her disease, which had been pronounced an advanced case of consumption by prominent physicians. This poor sufferer had not derived any benefit whatsoever from the treatment, and as a result her conscience would not permit her to become a partner to such a fraudulent procedure. Some of these unscrupulous concerns resort to absolute fraud to beguile the public by using the name of the great scientist and benefactor, Prof. Robert Koch, of Berlin, as though he were associated with them in their business and treatment. They advertised his picture beside that of an individual with a similar name, and are heading their advertisements as "Professor Robert Koch's Cure."

During the past year the fraud referred to by Dr. Knopf as Professor Robert Koch's Cure has been fully exposed in the New York *Herald* on evidence submitted by the Medical Society of the County of New York. From their own advertisement it will be seen that everywhere the impression is given that Dr. Edward Phillip Koch and Dr. Robert Koch are in professional relations with each other. Their so-called cure has offices in Buffalo,* Chicago, Boston, Pittsburg, Altoona, Philadelphia, Cincinnati, Baltimore, Washington, Rochester, St. Louis, Newark and other cities. Dr. Koch is advertised in connection with the various establishments. In the New York establishment the society has never yet been able to capture Dr. Edward Phillip Koch but he was recently brought to justice in the District of Columbia. He was, in May, 1904, convicted of practising medicine without registration.

Dr. Robert Koch, of Germany, has protested most bitterly against the use of his name by quacks and charlatans, and denying, of course, the slightest connection with any so-called cure or with any physician of a similar name anywhere in the world. As long as registered physicians can be induced to take part in such deception it will be difficult to close this establishment by law, but the conviction of Dr. Edward Phillip Koch and the wide publicity given to his methods will greatly lessen the dangers of such establishments.

So-called Specialist in "Diseases of Men."—Almost as dangerous is the so-called specialist in diseases of men. Certain newspapers in New York print columns of their advertisements without apology to the public for intruding such filthy printed matter. The methods of these quacks are various. Most of them advertise in the name of a registered physician and then employ green youths and unlicensed practitioners to meet the patient after he calls. As the law at present constituted does not require a physician employed in an advertising institution to make known his identity, it is difficult to trace out the identity of the practitioner in any of these concerns. The employer generally keeps them moving and does not permit a practitioner to remain in any one office long enough for very much information to be found out about him. Other offices advertise in the name of men not licensed to practice, but put in charge of their office in New York a licensed practitioner.

The writer is reliably informed that one institution of this character in New York City made over $200,000 last year. I know of several instances where by making a false diagnosis and frightening the patient into the belief that he had a loathsome disease, the patient has been induced to pay over as high as $2,000 for a few treatments. The law must be amended so as to compel every practitioner in an advertising institution to make known his identity, just as every dentist in any advertising dental parlor is required by law to post his name above the chair where he operates.

* The Buffalo joint has changed its methods to and name to the International Electro-Physical Institute, with the same proprietor.—Editor.

In brief, such is the work of the Medical Society of the County of New York. With its professed antagonism to so many powerful yet corrupt influences, it has an abundance of enemies, many of them occupying the seats of the mighty. In these enemies it finds the highest testimonial to the efficiency of its work and the greatest incentive to a continuance of its labors.

